# Which and How Many Patients Should Be Included in Randomised Controlled Trials to Demonstrate the Efficacy of Biologics in Primary Sjögren’s Syndrome?

**DOI:** 10.1371/journal.pone.0133907

**Published:** 2015-09-14

**Authors:** Valérie Devauchelle-Pensec, Jacques-Eric Gottenberg, Sandrine Jousse-Joulin, Jean-Marie Berthelot, Aleth Perdriger, Eric Hachulla, Pierre Yves Hatron, Xavier Puechal, Véronique Le Guern, Jean Sibilia, Laurent Chiche, Vincent Goeb, Olivier Vittecoq, Claire Larroche, Anne Laure Fauchais, Gilles Hayem, Jacques Morel, Charles Zarnitsky, Jean Jacques Dubost, Philippe Dieudé, Jacques Olivier Pers, Divi Cornec, Raphaele Seror, Xavier Mariette, Emmanuel Nowak, Alain Saraux

**Affiliations:** 1 Rheumatology Department, CHU de la Cavale Blanche, Boulevard Tanguy Prigent, 29609, Brest, France; 2 EA 2216, INSERM ESPRI, ERI29 Université Bretagne Occidentale, 29200, Brest, France; 3 Rheumatology Department, Strasbourg University Hospital, Strasbourg, France; 4 Rheumatology Department, Hôtel-Dieu, CHU Nantes, 44093, Nantes Cedex 01, France; 5 Rheumatology Department, C.H.U. Hôpital Sud, 35000, Rennes, France; 6 Internal Medicine Department, Claude Huriez Hospital, Lille2 University, 59037, Lille Cedex, France; 7 Internal Medicine Department, Hôpital Cochin, Paris, France; 8 Internal Medicine Department, Hôpital de la Conception, 147 Bd Baille, 13005, Marseille, France; 9 Rheumatology Department, C.H.R.U. d’Amiens, 76 230 Bois-Guillaume, France; 10 Rheumatology Department, C.H.R.U. de Rouen, 76 230 Bois-Guillaume, France; 11 Internal Medicine Department, Bobigny University Hospital, Paris, France; 12 Internal Medicine Department, Limoges University Hospital, Limoges, France; 13 Rheumatology Department, Ambroise Paré University Hospital, Paris, France; 14 Immuno-Rhumatology Department, C.H.U. Lapeyronie, 34295 Montpellier, France; 15 Rheumatology Department, C.H. J. Monod, Montivilliers, France; 16 Rheumatology Department, Gabriel Montpied Teaching Hospital, Place H. Dunant, Clermont-Ferrand, 63000, France; 17 Rheumatology Department, Bichat Claude-Bernard Hospital, Paris, France; 18 Rheumatology Department, Assistance Publique-Hôpitaux de Paris (AP-HP), INSERM U1012, Université Paris-Sud, 78 rue du Général Leclerc, 94275 Le Kremlin Bicêtre, France; 19 INSERM CIC 0502, CHU Brest, Brest, France; Università di Palermo, ITALY

## Abstract

**Objective:**

The goal of this study was to determine how the choice of the primary endpoint influenced sample size estimates in randomised controlled trials (RCTs) of treatments for primary Sjögren’s syndrome (pSS).

**Methods:**

We reviewed all studies evaluating biotechnological therapies in pSS to identify their inclusion criteria and primary endpoints. Then, in a large cohort (ASSESS), we determined the proportion of patients who would be included in RCTs using various inclusion criteria sets. Finally, we used the population of a large randomised therapeutic trial in pSS (TEARS) to assess the impact of various primary objectives and endpoints on estimated sample sizes. These analyses were performed only for the endpoints indicating greater efficacy of rituximab compared to the placebo.

**Results:**

We identified 18 studies. The most common inclusion criteria were short disease duration; systemic involvement; high mean visual analogue scale (VAS) scores for dryness, pain, and fatigue; and biological evidence of activity. In the ASSESS cohort, 35 percent of patients had recent-onset disease (lower than 4 years), 68 percent systemic manifestations, 68 percent high scores on two of three VASs, and 52 percent biological evidence of activity. The primary endpoints associated with the smallest sample sizes (nlower than 200) were a VAS dryness score improvement higher to 20 mm by week 24 or variable improvements (10, 20, or 30 mm) in fatigue VAS by week 6 or 16. For patients with systemic manifestations, the ESSDAI change may be the most logical endpoint, as it reflects all domains of disease activity. However, the ESSDAI did not improve significantly with rituximab therapy in the TEARS study. Ultrasound score improvement produced the smallest sample size estimate in the TEARS study.

**Conclusion:**

This study provides valuable information for designing future RCTs on the basis of previously published studies. Previous RCTs used inclusion criteria that selected a small part of the entire pSS population. The endpoint was usually based on VASs assessing patient complaints. In contrast to VAS dryness cut-offs, VAS fatigue cut-offs did not affect estimated sample sizes. SGUS improvement produced the smallest estimated sample size. Further studies are required to validate standardised SGUS modalities and assessment criteria. Thus, researchers should strive to develop a composite primary endpoint and to determine its best cut-off and assessment time point.

## Introduction

Primary Sjögren’s syndrome (pSS) is a chronic autoimmune disorder that induces dryness of the eyes (xerophthalmia) and mouth (xerostomia); salivary gland lesions; and presence of autoantibodies including anti-SSA, anti-SSB, and/or rheumatoid factor. The prevalence of pSS ranges from less than 0.1 to 1 percent [1], and adult women are predominantly affected. The manifestations are disabling symptoms due to ocular and oral dryness combined with fatigue [2] and severely impaired quality of life [3, 4]. Diffuse pain and fibromyalgia are also present in 5 percent of pSS patients [2, 5], as seen in systemic lupus erythematosus (SLE). In addition, 5 percent to 50 percent of patients have systemic manifestations consisting chiefly of rheumatologic, neurologic, pulmonary, hematologic, and renal disorders [6]. B-cell hyperactivity is the hallmark of the disease, and presence of germinal centres in the salivary glands predicts the development of lymphoma [7, 8]. Thus, the presentation of pSS varies to an extraordinary extent across patients and over time, and key symptoms are partly assessed using subjective tests. These characteristics of pSS raise major challenges when designing studies to assess treatment responses.

To date, no systemic treatment has been proven effective in altering the course of pSS [9]. The recent development of several monoclonal antibodies and new insights into the pathophysiology of pSS have provided opportunities for evaluating new treatment targets, leading to a dramatic increase in the number of randomised controlled trials (RCTs) in pSS. The first RCTs assessed TNF alpha antagonists and failed to demonstrate efficacy [10, 11]. More recently, studies focussed on B cells [12], which play a central role in the development of pSS [13–15], and on other targets such as interleukin-6 and CTLA-4 [16, 17]. Preliminary open-label studies of the safety and efficacy of biologics produced encouraging results [18–20] and were followed by small RCTs, which indicated efficacy in improving visual analogue scale (VAS) scores for fatigue and dryness, as well as stimulated whole salivary flow [21, 22]. However, in the TEARS trial [23] (Tolerance and EfficAcy of Rituximab in primary Sjögren’s syndrome), a large multicentre double-blind RCT in patients with active recent and/or systemic pSS, rituximab failed to significantly improve the primary endpoint versus a placebo at week 24, although a significant improvement was noted at week 6. Clearly, it would be useful to determine the minimal clinically important differences for endpoints used to evaluate treatments. There is also a need for estimating the sample sizes required for future studies of pSS according to the primary endpoint [24].

The goal of this study was to determine how the choice of the primary endpoint influenced sample size estimates in RCTs of treatments for pSS. We reviewed the inclusion criteria and primary endpoints used in published RCTs, and we analysed TEARS study results to evaluate how changes in these criteria and endpoints affected the sample size required for future RCTs.

## Patients and Methods

### Study Design

We reviewed the literature to identify the most widely used inclusion criteria for RCTs of biologics in pSS. We then applied those criteria to the ASSESS cohort [15], a recent prospective cohort of patients with well-established pSS, to determine the proportions of patients who would have been considered eligible for the RCTs. Finally, we conducted a post hoc analysis of TEARS trial data to evaluate how the primary endpoint affected the required sample size.

### Literature Review

We used MeSH terms to search PUBMED, EMBASE, and clinicaltrial.gov for trials of biologics in pSS published or registered between 2000 and 2014. We considered all trials for which the inclusion criteria and primary endpoint were clearly defined.

### Study Populations

We applied various inclusion criteria sets to the ASSESS (Assessment of Systemic Signs and Evolution in Sjögren’s Syndrome) cohort [15], a multicentre prospective cohort of patients with well-established pSS created in 2006 to identify factors predicting lymphoma during a 5-year prospective follow-up. All patients gave their written informed consent to participation in the study, which was approved by the appropriate ethics committee. To ensure that the study population would be representative of the entire population with pSS, consecutive patients fulfilling American-European Consensus Group (AECG) criteria for pSS were enrolled. Fifteen centres recruited 395 patients. At baseline, median (25(th)-75(th)) disease duration was 5 (2–9) years, median EULAR Sjögren’s Syndrome Disease Activity Index (ESSDAI) [25, 26] was 2 (0–7.0), and median EULAR Sjögren’s Syndrome Patient Reported Index (ESSPRI) [27] was 5.7 (4.0–7.0). Reported cryoglobulinaemia (17.0 percent of patients), IgG elevation (29.3 percent), and decreased C4 (19.4 percent). Autoantibodies (anti-SSA, anti-SSB, and rheumatoid factor) were present in 59.2 percent, 33.5 percent, and 41.1 percent of patients, respectively.

To evaluate the effect of the primary endpoint on the number of eligible patients, we performed a post hoc analysis of data from the TEARS study, a large double-blind RCT comparing the efficacy of rituximab to a placebo in pSS [23]. All patients met AECG criteria [28] and had active disease defined as values ≥50/100 mm for at least two of four VASs evaluating dryness, pain, fatigue, and global disease, respectively. Eligibility criteria were either recent disease (lower than 10 years since symptom onset) with biological activity (anti-SSA or rheumatoid factor) or cryoglobulinaemia or hypergammaglobulinaemia or elevated ß2-microglobulinaemia, or hypocomplementaemia; or systemic pSS defined as at least one extra-glandular manifestation. The primary endpoint was a 30-mm improvement from week 0 to week 24 on at least two of the four VAS scores. Secondary endpoints included improvement from baseline to week 24 in each of the four VAS scores, the ESSDAI [25, 26]; basal salivary flow rate; salivary-gland ultrasound (SGUS) grade [19]; Schirmer’s test results; van Bijsterveld scores; Chisholm grade; and laboratory variables (C-reactive protein and erythrocyte sedimentation rate; rheumatoid factor; antinuclear antibodies; serum IgG, IgA, and IgM levels; serum complement; cryoglobulinaemia; and serum level of B-cell-activating factor. A substudy was performed in a single centre, where 28 patients underwent B-mode and Doppler ultrasonography of the parotid and submandibular glands for assessments of echostructure and vascularisation. The 122 patients were recruited at 14 university hospitals and randomly assigned in a 1:1 ratio to blinded treatment with intravenous rituximab infusions (1 g) or placebo at weeks 0 and 2. Among them, 24 had recent-onset pSS, 31 systemic pSS, and 67 both.

### Statistical Analysis

We described the inclusion criteria and endpoints used in published RCTs of treatments for pSS. We then separated the ASSESS cohort patients using the S1 File into groups based on whether they met the main inclusion criteria used in published RCTs, to evaluate the proportion of patients who would have been considered eligible (see supporting informations).

We estimated the sample sizes required to obtain 80 percent power for detecting each of the TEARS study endpoints, using Epi Info 7 (method based on the Fleiss formula) [29] on the basis of the SAS data set of the S2 File with the imputed values used for the analysis in supporting informations (Labels are available in the SAS data set to understand variables names; the SAS code to find again the results; titles allow to understand which results are being talked about). These analyses were performed only for the endpoints indicating greater efficacy of rituximab compared to the placebo.

## Results

### Inclusion Criteria Used in Previous Studies

**Fig 1:**
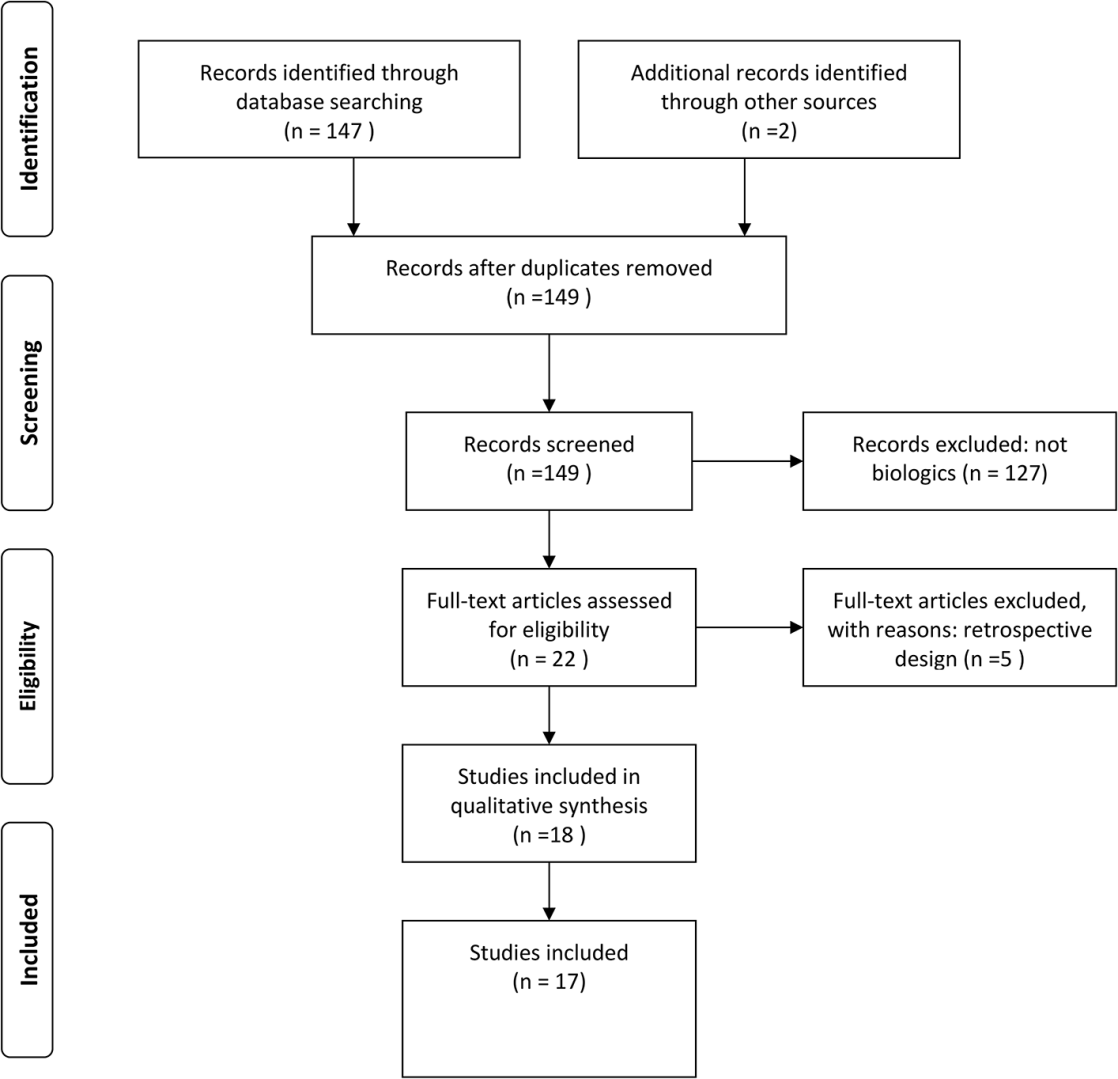
Flow diagram.

Of 147 publications identified on pubmed between 2001 and 2014 and two ongoing studies identified on clinicaltrial.gov. using “sjogren’s syndrome” within the limit “clinical trial”, we identified 17 studies evaluating any biologic in pSS (Fig 1).

Most of them evaluated TNF alpha antagonists, abatacept or rituximab [10, 11, 18–23, 30–38]. An open-label study tested epratuzumab in patients with B-cell overactivity, but no RCT is available for this drug. An open-label design was used in 8 studies (Table 1). Nine studies were published or ongoing RCTs (Table 2). One unblinded non-randomised trial evaluating the efficacy of rituximab versus synthetic disease-modifying antirheumatic drugs in two centres [30] was excluded due to the absence of randomisation. All preliminary open-label studies suggested efficacy of the evaluated biologics other than etanercept. Five open-label studies and three RCTs evaluated rituximab; another study of this drug is ongoing [31]. More recently, studies evaluated belimumab [32] and abatacept [18]. A large RCT is currently evaluating tocilizumab, for which no open-label data are available. All these studies used a combination of objective and subjective inclusion criteria. The most commonly used inclusion criteria were those in the AECG classification. In addition, 4 of the 8 open-label studies used the presence of autoantibodies, 3 the VAS scores, and 3 the systemic manifestations. In RCTs (Table 2), all inclusion criteria were based on the AECG classification, with salivary gland biopsy abnormalities, autoantibodies, or salivary flow rate impairment. Composite inclusion criteria were used in some RCTs; they were based on systemic manifestations in 5/9 studies, VAS score elevation in 5/9 studies, recent disease onset in 2/7 studies, and biological activity in 2/7 studies. Systemic manifestations have been used more often since 2011, probably due to the introduction of the ESSDAI.

**Table 1:** Open-label therapeutic trials of biologics in primary Sjögren’s disease reported between 2002 and 2014.

Reference	Inclusion criteria	Treatment andType of study	N	Primary endpoint	Primary endpoint achieved
[33, 34]	AECG *and* autoantibodies*and* active pSS	Infliximab single-centre, open-label	16	At weeks 2, 6, 10, or 14 Relapse defined as a 30% increase in symptoms of dry eyes, dry mouth, or fatigue, and/or a 30% increase in the ESR	NA
[11]	AECG *and* SGB *and* IgA plasmacellslower than70percent *and* moderate or severe fatigue	Etanercept single-centre, open-label	15	At weeks 4,8,12, 18, and 24 MFI questionnaire, VAS, serological monitoring, salivary flow tests, Schirmer’s test, Rose Bengal corneal stain, tear-film breakup, SGB	Exploratory No data suggesting efficacy
[20]	1-Early pSS (lower than 4 years): AECG *and*B cell overactivity (IgG higher to15 mg/L) and autoantibodies (IgM rheumatoid factor, anti-SSA/SSB). 2-MALT pSS group: AECG *and* localised MALT-type lymphoma (stage IE)	Rituximab Open-label	15	ExploratoryAt weeks 5 and 12 Immunologic markers, salivary/lacrimal functions, and subjective parameters MALT-type lymphoma was restaged 12 weeks after treatment initiation in the MALT/primary SS group.	Exploratory Data suggesting efficacy
[35]	Revised AECG *and* autoantibodies *and* biological activity (IgG higher to 1.4 g/L or ESR higher to 25 mm/h)	EpratuzumabOpen-labelTwo centres	16	Improvement ≥20 percent in at least 2/4 parameters: Schirmer’s test, unstimulated whole salivary flow, VAS for fatigue, and ESR, ± IgG level	
[19]	Revised AECG *and* active pSS (score higher to 50 on at least 2/4 VASs evaluating dryness, pain, fatigue, and global disease)	RituximabOpen-label	16	Exploratory At weeks 12, 24 and 36 Safety and clinical and biological parameters, SGB, SF-36 and SGUS	Exploratory Data suggesting efficacy
[32] BELISS	Revised AECG with anti-SSA *and* current systemic complications *or* recent disease (lower than5 years) *or* biomarkers of B-cell activation	BelimumabTwo centresOpen-label		At week 28 2 of 5 response criteria: Higher or equal 30 percent reduction in patient dryness VAS Higher or equal 30 percent reduction in patient fatigue VAS Higher or equal 30 percent reduction in patient musculoskeletal pain VAS Higher or equal 30 percent reduction in physician systemic activity VAS Higher or equal 25 percent reduction in serum levels of any of the following: B-cell activation biomarkers (free light immunoglobulin chains, Béta2-microglobulin, monoclonal component, cryoglobulinaemia, IgG) or higher or equal 25 percent C4 increase	Exploratory Data suggesting efficacy
[36]	AECG *and* one or more severe disease manifestations, including fatigue(VAS higher to 50 mm), joint pain (VAS higher to 50 mm), parotid glandswelling, *or* other systemicmanifestations	Rituximab Open-label	12	At week 26 Safety and clinical and biologic activity	Exploratory No data suggesting clinical efficacy
[18]	AECG*and* recent disease (lower or equal 5 years) *and* stimulatedwhole saliva higher or equal 0.10 mL/min *and* autoantibodies (RF, anti-SSA, or anti-SSB)*and* abnormal SGB	AbataceptSingle-centre open-label	15	ExploratoryWeeks 4, 12, 24 (on treatment), ESSDAI and ESSPRIat weeks 36 and 48, ESSPRI	Exploratory Data suggesting efficacy

N, number of patients; pSS, primary Sjögren’s syndrome; AECG: American-European Consensus Group criteria for primary Sjögren’s syndrome; SGB, salivary-gland biopsy; Ig, immunoglobulin; MALT, mucosa-associated lymphoid tissue; ESR, erythrocyte sedimentation rate; VAS, visual analogue scale (0–100 mm); RF, rheumatoid factor; MFI, Multidimensional Fatigue Inventory; C4, fraction 4 of complement; ESSDAI, European League Against Rheumatism (EULAR) Sjögren's Syndrome Disease Activity Index; ESSPRI, EULAR Sjögren's Syndrome Patient Reported Index; SF-36, Short-Form 36-item quality of life questionnaire; SGUS, salivary-gland ultrasound; NA, not applicable; BELISS: Belimumab in primary Sjögren’s Syndrome

**Table 2:** Controlled therapeutic trials of biologics in primary Sjögren’s syndrome reported between 2002 and 2014.

Reference	Inclusion criteria	Treatment andType of study	N	Primary endpoint	Significant difference for primary endpoint
[37]	AECG *and* oral *and* ocular dryness *and* activepSS (ESR or IgG levels)	EtanerceptDouble-blind, placebocontrolled	14	Higher or equal 20% improvement from baseline for 2 of 3 domains: subjective or objective measures of dry mouth and dry eyes, and IgG level or ESR	No
[10] TRIPPS	AECG *and* active pSS with 2/3 VAS higher to 50 mm(pain, fatigue, and the most disturbing dryness)	Infliximab Multicentre, placebo-controlled, double-blind	103	At week 10 Higher or equal 30% improvement in 2 of 3 VASs measuring joint pain, fatigue, and the most disturbing dryness.	No and no differences for secondary outcomes
[21]	Revised AECG*and* VAS fatigue higher to 50 mm	Rituximab Placebo-controlled Double-blind	17	At week 24 20 percent reduction in VAS fatigue score	Yes
[22]	AECG *and*stimulated whole saliva higher or equal 0.15mL/minute *and* autoantibodies (IgM-RF and anti-SSA and/or anti-SSB) *and* SGB grade III or IV	Rituximab Placebo-controlled Double-blind	30	At weeks 5, 12, 24, and 48 Improvement in the stimulated whole saliva flow rate	Yes Significant improvement at weeks 5 and 12
[23] TEARS	AECG *and* recent disease (lower than10 years) with biological activity *or* systemic manifestations *and* 2 of 4 VAS higher to 50 mm (global disease, pain, fatigue and dryness)	Rituximab Prospective Placebo-controlled Double-blind Multicentre	122	At week 24 30-mm improvement in 2 of 4 VASs	No but efficacy on secondary endpoints
[38]	AECG With fatigue measured by the Fatigue Severity Scale (FSS) (score lower or equal 3)	Anakinra Randomised Placebo-controlled Double-blind	26	Fatigue scores at week 4 adjusted for baseline values.	*No*
[31] TRACTISS ongoing	AECG with VAS for fatigue and oral dryness higher or equal 50 mm *and* anti-Ro antibodies *and* unstimulated salivary flow rate higher to 0 mL/min. With systemic involvement if disease duration higher to 10 years	Rituximab Randomised Placebo-controlled Double-blind Multicentre	110	At 48 weeks 30 percent improvement in VAS fatigue or oral drynessscore	*ongoing*
Clinicaltrial.gov OngoingBootsma et al	AECG and ESSDAI higher or equal 5	Abatacept Randomised Placebo-controlled Double-blind	88	At week 24ESSDAI	*ongoing*
Clinicaltrial.gov ETAPOngoingGottenberg et al.	AECG *and* anti-SSA or anti-SSB *and* ESSDAI higher or equal 5	TocilizumabRandomisedPlacebo-controlledDouble-blindMulticentre	120	At week 24ESSDAI improvement ≥3 points versus baseline	*ongoing*

TRIPPS: Trial of Remicade In Primary Sjogren's Syndrome; TEARS: Tolerance and EfficAcy of Rituximab in primary Sjögren syndrome; TRACTISS, Trial of Anti-B-Cell Therapy In primary Sjögren’s Syndrome; ETAP: Efficacy of Tocilizumab in Primary Sjögren's Syndrome; AECG, American-European Consensus Group criteria for primary Sjögren’s syndrome; ESR, erythrocyte sedimentation rate; Ig, immunoglobulin; pSS, primary Sjögren’s syndrome; VAS, visual analogue scale (0–100 mm); RF, rheumatoid factor; SGB: salivary gland biopsy; ESSDAI, European League Against Rheumatism (EULAR) Sjögren's Syndrome Disease Activity Index; N, number of patients.

In summary, the main inclusion criteria were a short disease duration (<4, 5, or 10 years); systemic involvement (ESSDAIhigher to 1); VAS scores higher to 5/10 for dryness, pain, and fatigue; and biological activity markers (hypergammaglobulinaemia and/or cryoglobulinaemia, and/or high béta 2 microglobulinaemia, and/or low C4). None of the studies assessed the presence of germinal centres or number of foci in salivary-gland biopsies [39, 40] or the SGUS [41].

### Application of Inclusion Criteria to the ASSESS Cohort

**Fig 2:**
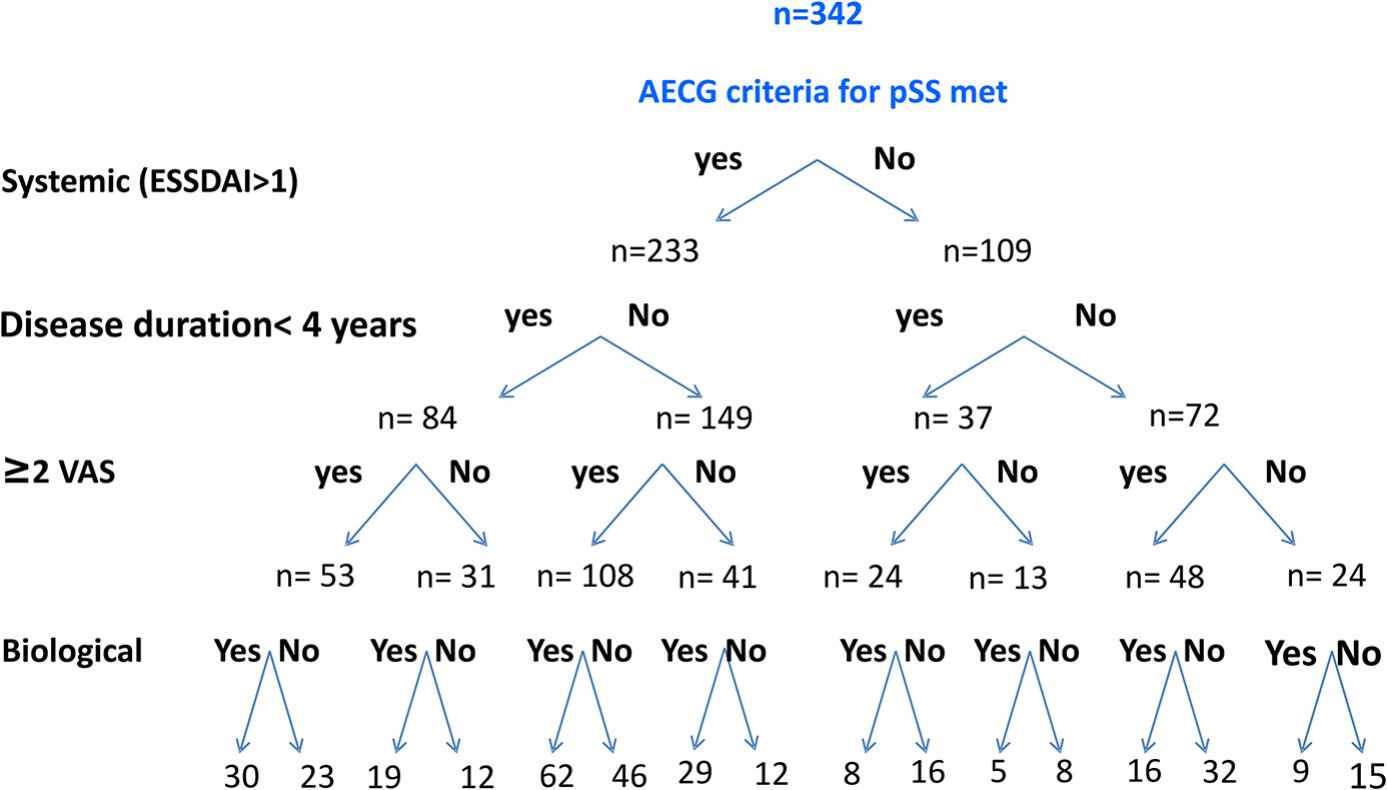
Number of patients in the ASSESS cohort fulfilling each inclusion criteria set. First step: AECG criteria. Second step: systemic disease (ESSDAIhigher to 2). Third step: disease duration less than 4 years. Fourth step: More than two VAS/3 higher to 50/100 mm. Fifth step: biological activity (pSS, primary Sjögren’s syndrome; AECG, American-European Consensus Group; ESSDAI, EULAR Sjögren Syndrome Disease Activity Index; VAS, visual analog scale (100-mm line); BA, biological activity).

Of the 395 patients included in the ASSESS cohort, 342 (87 percent) had the data needed to assess the presence of the main inclusion criteria used in studies of biologics in pSS. At least two VAS scores were higher to 50/100 in 233/342 (68 percent) patients, and 232 (68 percent) patients had systemic manifestations with an ESSDAI ≥2 (Fig 2). Only 35 percent (121/342) of patients had recent-onset disease. Requiring symptom onset within the last 4 years, systemic disease, at least two of three VAS scores higher to 50/100, and biological activity would result in the inclusion of only 30/342 (9 percent) patients. The combination of recent-onset or systemic pSS with at least two of three VAS scores higher to 50/100 and biological activity would include 100/342 (29 percent) patients.

### Sample Sizes According to TEARS Study Endpoints

In the TEARS study [23], one primary and several secondary endpoints were used to compare the efficacy of rituximab and of a placebo. The primary endpoint was an at least 30-mm improvement at week 24 in at least two of four VAS scores for fatigue, dryness, pain, and global disease. The proportion of patients who achieved the primary endpoint was not significantly different between the rituximab and placebo groups. However, several other endpoints were significantly better with rituximab. Thus, at least two of three VAS scores improved by more than 30 mm by week 6. The VAS dryness score was significantly improved at week 24 and the SGUS score was improved at week 24. Higher proportions of improved patients were found in the rituximab group for the VAS fatigue and global disease scores and for the ESSPRI. We computed the proportions of patients with improvements in the four VAS scores and in the ESSPRI or SGUS score defined using various cut-offs, and we determined the sample sizes required to demonstrate differences during future RCTs at weeks 6, 16, and 24 (Table 3). At week 24, the greatest difference between the placebo and rituximab groups was for the VAS dryness score. Using cut-offs of VAS changes of 10, 20, or 30 mm induced large changes in the estimated sample size. A cut-off higher to 20 mm increased the sample size from 132 to more than 300 at week 24 compared to a cut-off of 10 mm. In contrast, the VAS fatigue score cut-off did not influence sample size, which was less than 200 for assessment at week 6 or 16. An at least 10-point improvement in the ESSPRI was achieved by 340 patients at week 24, but detecting a larger improvement would have required over 1000 patients. SGUS was associated with the smallest sample size required to detect an effect of rituximab versus placebo. Nevertheless, in the TEARS study, the SGUS score change was not associated with the improvement in the mean VAS dryness score from baseline to week 24.

**Table 3:** Number of patients to include in future randomised controlled trials of rituximab in primary Sjögren’s syndrome according to the primary endpoint assessed at weeks 6, 16, and 24.

**Patient improvement**	**At week 6, P vs. R; N: sample size**			**At Week 16, P vs. R; N: sample size**			**At week 24, P vs. R; N: sample size**		
**VAS scores**	higher or equal **10 mm**	**higher or equal 20 mm**	**higher or equal 30 mm**	**higher or equal 10 mm**	**higher or equal 20 mm**	**higher or equal 30 mm**	**higher or equal 10 mm**	**higher or equal 20 mm**	**higher or equal 30 mm**
Disease	33.8 vs. 43.8 N = 782	18.0 vs. 31.7 N = 338	8.0 vs. 15.8 N = 588	34.0 vs 53.3 N = 226	21.2 vs 33.4 N = 448	18.2 vs 20.5 N = 9432	35.8 vs 52.7 N = 292	26.3 vs 36.0 N = 754	24.0 vs 16.9 N = NA
Pain	34.1 vs. 34.4 N higher to 10000	25.5 vs. 28.7 N = 6180	14.0 vs. 18.0 N = 2734	30.1 vs 34.6 N = 3478	15.8 vs 21.8 N = 1394	15.9 vs 15.2 N = NA	39.2 vs 44.1 N = 3256	33.0 vs 25.7 N = NA	22.0 vs 12.6N = NA
Fatigue	30.8 vs. 54.7 N = 148	17.1 vs. 39.4 N = 142	8.2 vs. 34.7 N = 88	21.9 vs 47.0 N = 126	15.0 vs 38.7 N = 124	8.9 vs 27.2 N = 158	31.2 vs 51.5 N = 202	17.8 vs 29.2 N = 466	10.8 vs 20.1 N = 514
Dryness	25.3 vs. 45.7 N = 190	13.0 vs. 29.9 N = 206	8.6 vs. 16.6 N = 586	32.1 vs 53.9 N = 178	16.5 vs 26.2 N = 598	13.6 vs 21.1 N = 850	26.3 vs 51.3 N = 132	17.2 vs 31.0 N = 328	13.2 vs 25.6 N = 348
**ESSPRI**	33.3 vs. 49.2 N = 324	21.6 vs. 32.5 N = 556	7.3 vs. 22.5 N = 196	24.2 vs 41.0 N = 266	16.7 vs 27.5 N = 498	10.2 vs 13.5 N = 3130	29.1 vs 44.3 N = 340	22.2 vs 28.9 N = 1388	13.0 vs 13.1 N higher to 10000
**Patient improvement**	**At Week 6, P vs. R; N: sample size**			**At Week 16, P vs. R; N: sample size**			**At week 24, P vs. R; N: sample size**		
**Number of VAS scores improved by 30 mm (at least)**	**higher or equal 1**	**higher or equal 2**	**higher or equal 3**	**higher or equal 1**	**higher or equal 2**	**higher or equal 3**	**higher or equal 1**	**higher or equal 2**	**higher or equal 3**
	26.7 vs. 49.5N = 158	9.1 vs. 22.4 N = 262	3.1 vs. 11.1 N = 370	24.5 vs 50.0N = 126	17.0 vs 26.3 N = 656	10.1 vs 6.5 N = NA	37.8 vs 42.2 N = 3980	22.0 vs 23.0 N higher to 10000	11.9 vs 9.6 N = NA
**Improvement in SGUS grade**	**higher or equal 1 grade**			**higher or equal 1 grade**			**higher or equal 1 grade**		
	ND			ND			1/14 (7.1 percent) vs. 7/14 (50.0 percent) N = 42		

VAS, visual analogue scale; P, placebo; R, rituximab; ESSPRI, EULAR Sjögren’s Syndrome Patient Reported Index; SGUS: Salivary Gland Ultrasound; NA, not applicable; ND, not done

For patients with systemic manifestations, the ESSDAI change may be the most logical endpoint, as it reflects all domains of disease activity. However, the ESSDAI did not improve significantly with rituximab therapy in the TEARS study.

## Discussion

Because pSS is a complex and heterogeneous disease that is difficult to evaluate objectively, measuring treatment responses is challenging. Validated endpoints are lacking. However, tools have been developed recently to assess systemic activity (ESSDAI) and burden to the patient (ESSPRI). The best time point for such assessments is unclear, and the inclusion criteria that should be used for clinical trials are debated. Answers to these issues are urgently needed to allow the design of feasible RCTs capable of providing clinically relevant information on the efficacy of treatments for pSS. We sought to obtain such answers by examining data from earlier studies.

Most of the open-label studies of biologics in pSS suggested good safety and efficacy, but these results were not confirmed in RCTs. Inadequate sample size may have prevented the RCTs from detecting significant efficacy and discordant results have been found between RCTs with large and small sample sizes. The use of classification criteria for patient selection is not sufficient: patients selected for RCTs should have manifestations for which improvement or stabilisation is feasible. In addition, they should exhibit biological markers of disease activity and disabling symptoms such as dryness, pain, and fatigue, since the vast majority of pSS patients report discomfort related to such symptoms. However, even when evaluated using the ESSPRI, these subjective symptoms may fail to correlate with objective tests and fluctuate over time. Furthermore, the relative contributions of active disease processes and irreversible residual damage to the symptoms may be difficult to determine. Given the lack of specificity of dryness and fatigue, most studies relied on composite criteria that included autoantibodies or salivary-gland biopsy abnormalities [3]. Systemic manifestations were rarely used as inclusion criteria in the past, probably due to the absence of a scoring system; since the introduction in 2011 of the ESSDAI, a mean ESSDAI higher to 5 has been used. In the TEARS study, rituximab failed to significantly improve the systemic manifestations compared to the placebo. In another study of 20 patients given rituximab and compared to 10 patients given a placebo [42], rituximab substantially improved the ESSDAI standardised response mean at week 24. Results of ongoing RCTs will probably provide more accurate results [31].

When we applied the most widely used inclusion criteria to a large nationwide cohort of patients with recent pSS, we found that most patients reported severe discomfort, with at least two of four VAS scores (dryness, fatigue, pain, and global disease) higher to 50/100. However, less than 20 percent of patients had the combination of recent-onset active disease with high VAS scores and biological activity. This point may affect the feasibility of RCTs. Only half the patients with disease onset within the last 4 years had evidence of biological activity. The ESSDAI was elevated, but the mean value was less than 2 points. Thus, the inclusion of patients with high sub-scores on a single ESSDAI domain may require international multicentre patient recruitment. Recent disease onset and/or systemic disease are widely believed to predict a better treatment response, particularly to biologics, compared to long-standing disease. Using these two inclusion criteria dramatically decreases the required sample size. On the opposite, most patients had at least two VAS scores greater than 50/100 and biological activity.

Efficacy data from therapeutic trials depend in large part on the primary endpoint. In the TEARS study, rituximab improved the VAS dryness and fatigue scores, in keeping with earlier findings [21, 22]. The definition of a clinically significant improvement in pSS is a current focus of research [42, 43]. Improvements in VAS scores were used in previously published studies [21, 22]. VAS fatigue score cut-offs of 10, 20, and 30 mm at week 6 or 16 were associated with similar sample size requirements. A 20-mm cut-off may provide a good balance between clinical significance and sample size. Rituximab is the first treatment for which an effect on incapacitating fatigue has been demonstrated using a randomised controlled design. For the VAS dryness score, choosing a cut-off greater than 10 mm dramatically decreased the sample size. For the ESSPRI, at least 150 patients would be needed regardless of the cut-off used.

The SGUS score deserves consideration as an endpoint. In the TEARS study, the parotid gland score improved significantly with rituximab therapy [44]. This endpoint produces the smallest sample size. SGUS is a simple and inexpensive procedure that can be repeated easily and safely over time and that is undergoing validation [45, 46]. However, the exact significance of the SGUS score in terms of the disease process is still being evaluated, and there is no published evidence that it correlates with improvements in salivary flow rate or VAS dryness scores.

In conclusion, this study provides valuable information for designing future RCTs on the basis of previously published studies. The most common inclusion criteria were short disease duration; systemic involvement; high mean VAS scores for dryness, pain, and fatigue; and biological evidence of activity. Combining these criteria would select only a small proportion of patients with pSS. The primary endpoints associated with the smallest sample sizes are a VAS dryness score improvement higher to 20 mm by week 24 and variable improvements (10, 20, or 30 mm) in the VAS fatigue score by week 6 or 16. SGUS improvement produced the smallest estimated sample size (n = 42). Further studies are required to validate standardised SGUS modalities and assessment criteria. Thus, researchers should strive to develop a composite primary endpoint and to determine its best cut-off and assessment time point. The Sjogren Syndome Responder Index (SSRI), recently published [47], could be a candidate.

## Supporting Information

S1 File S1 file was used to separate the ASSESS cohort patients into groups based on whether they met the main inclusion criteria used in published RCTs, to evaluate the proportion of patients who would have been considered eligible. (XLS)

S2 File S2 file SAS data set with the imputed values was used for the analysis (Labels are available in the SAS data set to understand variables names; the SAS code to find again the results; titles allow to understand which results are being talked about). (ZIP)
